# ANT(9)-Ic, a Novel Chromosomally Encoded Aminoglycoside Nucleotidyltransferase from Brucella intermedia

**DOI:** 10.1128/spectrum.00620-23

**Published:** 2023-04-11

**Authors:** Xiusheng Sheng, Wei Lu, Aifang Li, Junwan Lu, Chunhan Song, Jiefeng Xu, Youming Dong, Chunqing Fu, Xi Lin, Mei Zhu, Qiyu Bao, Kewei Li

**Affiliations:** a Medical Molecular Biology Laboratory, School of Medicine, Jinhua Polytechnic, Jinhua, China; b Institute of Biomedical Informatics, Key Laboratory of Medical Genetics of Zhejiang Province, Key Laboratory of Laboratory Medicine, Ministry of Education, School of Laboratory Medicine and Life Sciences, Wenzhou Medical University, Wenzhou, China; c Fifth Affiliated Hospital, Wenzhou Medical University, Lishui, Zhejiang, China; d Department of Clinical Laboratory, Zhejiang Hospital, Hangzhou, Zhejiang, China; Johns Hopkins University School of Medicine

**Keywords:** ANT(9)-Ic, aminoglycoside resistance, aminoglycoside-modifying enzyme, aminoglycoside nucleotidyltransferase, *Brucella intermedia*

## Abstract

Aminoglycoside-modifying enzymes are among the most important mechanisms of resistance to aminoglycoside antibiotics, typically conferring high-level resistance by enzymatic drug inactivation. Previously, we isolated a multidrug-resistant Brucella intermedia strain ZJ499 from a cancer patient, and whole-genome sequencing revealed several putative novel aminoglycoside-modifying enzyme genes in this strain. Here, we report the characterization of one of them that encodes an intrinsic, chromosomal aminoglycoside nucleotidyltransferase designated ANT(9)-Ic, which shares only 33.05% to 47.44% amino acid identity with the most closely related ANT(9)-I enzymes. When expressed in Escherichia coli, ANT(9)-Ic conferred resistance only to spectinomycin and not to any other aminoglycosides tested, indicating a substrate profile typical of ANT(9)-I enzymes. Consistent with this, deletion of *ant(9)-Ic* in ZJ499 resulted in a specific and significant decrease in MIC of spectinomycin. Furthermore, the purified ANT(9)-Ic protein showed stringent substrate specificity for spectinomycin with a *K_m_* value of 44.83 μM and a *k*_cat_/*K_m_* of 2.8 × 10^4^ M^−1^ s^−1^, echoing the above observations of susceptibility testing. In addition, comparative genomic analysis revealed that the genetic context of *ant(9)-Ic* was conserved in Brucella, with no mobile genetic elements found within its 20-kb surrounding region. Overall, our results demonstrate that ANT(9)-Ic is a novel member of the ANT(9)-I lineage, contributing to the intrinsic spectinomycin resistance of ZJ499.

**IMPORTANCE** The emergence, evolution, and worldwide spread of antibiotic resistance present a significant global public health crisis. For aminoglycoside antibiotics, enzymatic drug modification is the most common mechanism of resistance. We identify a novel chromosomal aminoglycoside nucleotidyltransferase from B. intermedia, called ANT(9)-Ic, which shares the highest identity (47.44%) with the previously known ANT(9)-Ia and plays an important role in spectinomycin resistance of the host strain. Analysis of the genetic environment and origin of *ant(9)-Ic* shows that the gene and its surrounding region are widely conserved in Brucella, and no mobile elements are detected, indicating that ANT(9)-Ic may be broadly important in the natural resistance to spectinomycin of Brucella species.

## INTRODUCTION

Aminoglycosides are highly potent antibiotics with broad-spectrum bactericidal activity and have been used in clinical practice for over 80 years (1, 2). In the past decades, the use of aminoglycoside antibiotics has been compromised due to the availability of other antibiotic classes and an increasing prevalence of resistance (3, 4). However, recent advances in understanding the molecular basis of aminoglycoside resistance have promoted the development of effective inhibitors and novel semisynthetic aminoglycosides such as arbekacin, plazomicin, and spectinamides (5), which in turn has spurred a resurgence of interest in this class of compounds (1).

The most prevalent mechanism of clinically relevant resistance against aminoglycosides is the enzymatic modification of the drug (6, 7). This process is catalyzed by three main classes of aminoglycoside-modifying enzymes (AMEs), including *N*-acetyltransferases (AACs), *O*-phosphotransferases (APHs), and *O*-nucleotidyltransferases (ANTs) (2, 4). The ANT class of enzymes mediate adenylation by transferring an AMP group from the donor ATP to a hydroxyl group of the aminoglycoside substrate (2). To date, five subclasses of ANTs containing over 40 distinct enzymes have been defined: ANT(2″), ANT(3″), ANT(4′), ANT(6), and ANT(9) (https://card.mcmaster.ca/ontology/36357). For the ANT(9) subclass, there are only two ANT(9)-I enzymes described in the literature: ANT(9)-Ia [also called Aad(9) or Spc] (8) and ANT(9)-Ib (9); both mediate specific resistance to spectinomycin (2). However, two other spectinomycin adenyltransferases, Spd (10) and Spw (11), which were named by another nomenclature system (12) after Spc, exhibit a resistance profile identical to the ANT(9)-I enzymes, suggesting that they also belong to the ANT(9)-I subtype. Notably, these enzymes are all encoded on plasmids or transposable elements, which has resulted in their rapid spread within a given species or among different bacterial species (2, 10, 11).

Brucella intermedia (previously known as Ochrobactrum intermedium) (13) is a Gram-negative bacillus that inhabits diverse terrestrial and aquatic environments and is considered to be an opportunistic human pathogen due to its ability to cause infections in both immunocompromised and immunocompetent hosts (14, 15). In a previous study, we reported the results of a genome-based screening for antibiotic resistance genes in a clinical isolate of B. intermedia (16). Here, we describe the analysis of one of those putative resistance determinants, designated *ant(9)-Ic*. We demonstrate the encoded protein, ANT(9)-Ic, to be a new member of the ANT(9)-I lineage by gene expression, inactivation, and characterization of the enzymatic properties.

## RESULTS AND DISCUSSION

### Identification of a novel member of the ANT(9)-I subclass, ANT(9)-Ic.

In a recent study, we isolated a multidrug-resistant B. intermedia strain ZJ499 from the sputum of a cancer patient at a university hospital in Lishui, China (16). This strain possessed broad-spectrum resistance to most β-lactams, aminoglycosides, polymyxins, phenicols, and diaminopyrimidines (16). To investigate the resistance mechanisms of ZJ499, we have previously conducted whole-genome sequencing and identified several potentially novel antibiotic resistance genes in this isolate, including those predicted to provide resistance to aminoglycosides (for additional information, see reference 16). Here, we describe the analysis of one of those putative aminoglycoside resistance determinants, an *ant(9)-I-like* gene (16), here designated *ant(9)-Ic* (GenBank accession no. MZ241296) in more detail.

**FIG 1:**
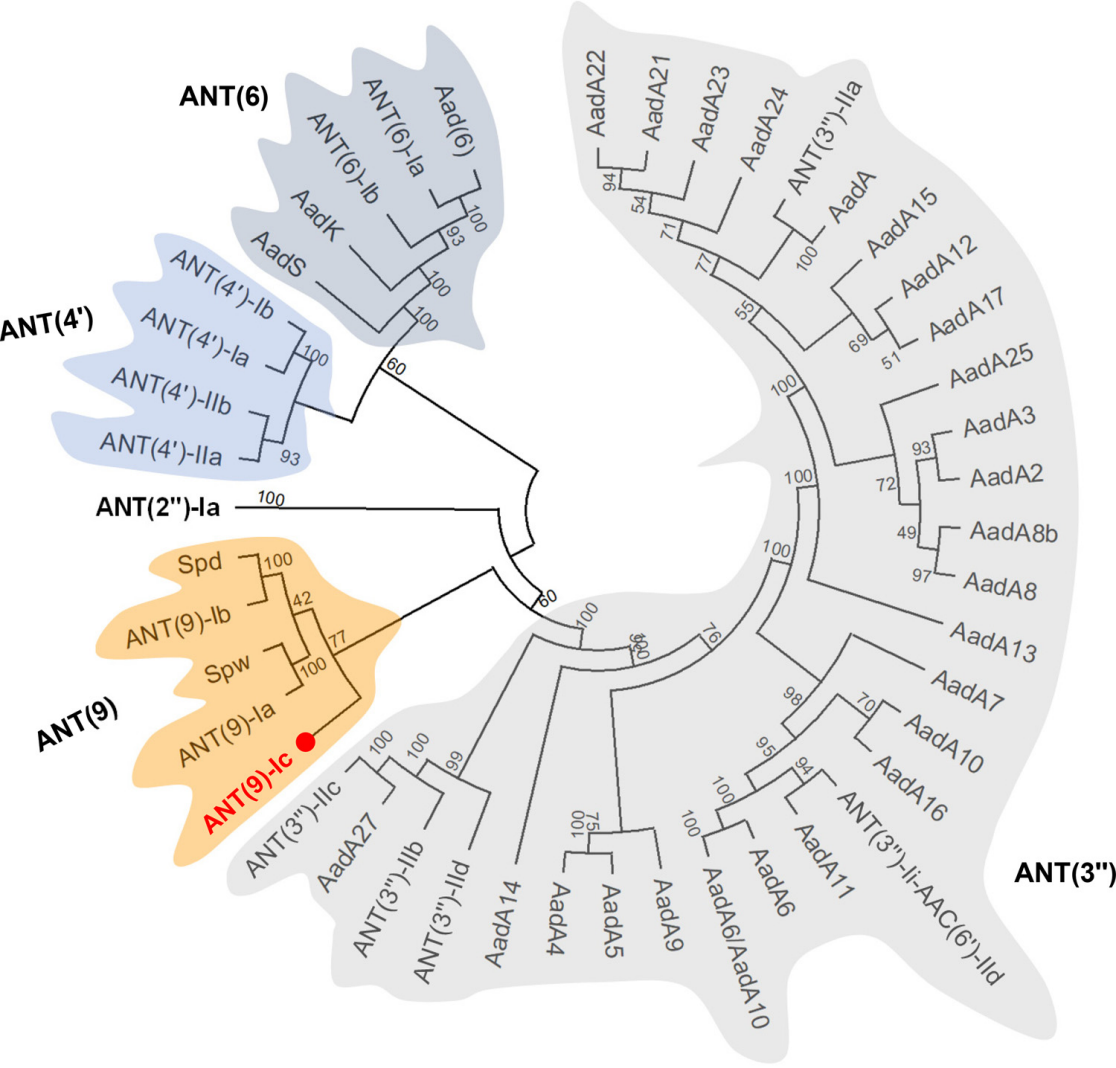
Phylogenetic analysis of ANT(9)-Ic and all other functionally characterized ANTs. The five distinct phylogenetic groups indicated with different background refer to ANT(9), ANT(2″), ANT(4′), ANT(6), and ANT(3″). Bootstrap values are shown at each node of the tree. ANT(9)-Ic from this study is highlighted in red with a red filled circle. The genBank accession numbers of the 45 proteins are as follows: ANT(9)-Ic, MZ241296; ANT(9)-Ia, CAA26963; Spw, EGP12870; ANT(9)-Ib, AAA16527; Spd, AGW81558; ANT(2″)-Ia, AAC64365; ANT(4′)-IIa, AAA25717; ANT(4′)-IIb, AAM76670; ANT(4′)-Ia, AAO83986; ANT(4′)-Ib, ADA62098; AadS, AAA27459; AadK, CAB14620; ANT(6)-Ib, CBH51824; ANT(6)-Ia, AHE40557; Aad(6), AAU10334; AadA22, CAK12750; AadA21, AAN87151; AadA23, CAH10847; AadA24, ABG72894; ANT(3″)-IIa, CAA26199; AadA, AAO49597; AadA15, ABD58917; AadA12, ACJ47200; AadA17, ACK43806; AadA25, AET15272; AadA3, AAC14728; AadA2, AAF27727; AadA8b, CAJ13568; AadA8, AAN41439; AadA13, ABW91178; AadA7, BAD00739; AadA10, AAL36430; AadA16, ACF17980; ANT(3″)-Ii-AAC(6′)-IId, AAL51021; AadA11, AAV32840; AadA6, CAJ32504; AadA6/AadA10, CAJ32491; AadA9, ABG49324; AadA5, AAF17880; AadA4, AAN34365; AadA14, CAI57696; ANT(3″)-IId, MW984426; ANT(3″)-IIb, ENU91137; AadA27, CTQ57092; and ANT(3″)-IIc, ENU37733.

The *ant(9)-Ic* gene is 780 bp in length, corresponding to a 259-amino acid protein with a calculated mass of 27,922 Da and a theoretical pI of 4.96. BLASTp search against the NCBI nr database using the amino acid sequence of ANT(9)-Ic returned 10 hits of >80% identity, which were all from Brucella. The top hit was a putative aminoglycoside nucleotidyltransferase ANT(9) (accession no. WP_006466136.1) from B. intermedia, with an amino acid sequence identity of 98.07% and query coverage of 100%. Phylogenetic analysis of ANT(9)-Ic and all previous functionally characterized ANTs collected from the NCBI nr and CARD databases showed that this protein clustered closest to the ANT(9) enzymes (Fig. 1), indicating that this protein belongs to the ANT(9) lineage. Within this group, ANT(9)-Ic exhibited the highest similarity (47.44% sequence identity) with ANT(9)-Ia (CAA26963) from Staphylococcus aureus (2, 8), and 34.18% identity with ANT(9)-Ib (AAA16527) from Enterococcus faecalis (9). Moreover, ANT(9)-Ic shared 33.05% identity with Spd (AGW81558) from S. aureus (10) and 42.19% sequence identity with Spw (EGP12870) from Lactobacillus johnsonii (11). From an evolutionary point of view, the low percentage of sequence homology between these subfamily enzymes might suggest an independent evolution or an evolutionary divergence from a common ancestor. Importantly, however, ANT(9)-Ic was detected using InterProScan to possess the conserved nucleotidyltransferase NT_KNTase_like and DUF4111 domains (10), which is consistent with the observation of other ANT(9)-I enzymes. Taken together, the features described above distinguish ANT(9)-Ic from other ANTs and are in favor of its nomenclature and classification.

### Confirmation of *ant(9)-Ic* as a spectinomycin resistance determinant.

To evaluate the resistance function of ANT(9)-Ic, we cloned *ant(9)-Ic* open reading frame (ORF) together with its promoter region into a multicopy plasmid pUCP20 in the laboratory Escherichia coli DH5α strain. As a control, we also included *ant(9)-Ib*, which is known to encode a functional spectinomycin-specific adenyltransferase (9, 17). In accordance with this description, expression of *ant(9)-Ib* increased the resistance of DH5α to spectinomycin by 64-fold compared to the control (DH5α/pUCP20), while the MICs of streptomycin and other 11 aminoglycosides tested remained unchanged (Table 1). Similar to this scenario, *ant(9)-Ic* mediated an increase only in spectinomycin resistance (Table 1), although the degree of increase (16-fold) was slightly lower than that of *ant(9)-Ib*. These data represent a functional proof and suggest that the substrate profile of ANT(9)-Ic corresponds to that of other ANT(9)-I-type enzymes.

**TABLE 1:** Functional analysis of ANT(9)-Ib, ANT(9)-Ic expressed in E. coli DH5α and resistance levels of WT B. intermedia ZJ499, as well as its *ant(9)-Ic*-deficient mutant to various aminoglycosides^*a*^

Strains	MIC (mg/liter)												
	SPE	STR	KAN	NEO	PAR	RIB	TOB	SIS	NET	APR	GEN	AMK	MCR
DH5α/pUCP20	8	2	2	1	1	2	0.25	0.25	0.25	2	0.25	1	0.25
DH5α/pUCP20-*ant(9)-Ib*	512	2	2	1	1	2	0.25	0.25	0.25	2	0.25	1	0.25
DH5α/pUCP20-*ant(9)-Ic*	128	2	2	1	1	2	0.25	0.25	0.25	2	0.25	1	0.25
B. intermedia ZJ499 (WT)	512	16	512	64	64	2,048	32	32	64	64	2	16	8
B. intermedia ZJ499 [Δ*ant(9)-Ic*]	32	16	512	64	64	2,048	32	32	64	64	2	16	8

a AMK, amikacin; APR, apramycin; GEN, gentamicin; KAN, kanamycin; MCR, micronomicin; NEO, neomycin; NET, netilmicin; PAR, paromomycin; RIB, ribostamycin; SIS, sisomicin; SPE, spectinomycin; STR, streptomycin; TOB, tobramycin; WT, wild type.

### ANT(9)-Ic provide spectinomycin resistance in its natural host.

Our previous work showed that the natural host (B. intermedia ZJ499) of *ant(9)-Ic* exhibited extensive resistance to aminoglycosides, including a high level of resistance to spectinomycin (16). To assess the contribution of *ant(9)-Ic* to the aminoglycoside resistance of ZJ499, we constructed a strain in which the coding region of *ant(9)-Ic* was completely deleted, and the Δ*ant(9)-Ic* mutant was tested for its susceptibility to various aminoglycosides. Compared to wild-type (WT) ZJ499, the Δ*ant(9)-Ic* mutant showed a 16-fold drop in spectinomycin resistance (Table 1), while the MICs of other aminoglycosides for Δ*ant(9)-Ic* were exactly the same as those observed for WT. These results suggest that ANT(9)-Ic contributes significantly to the intrinsic resistance of B. intermedia ZJ499 to spectinomycin and agree well with the resistance profile obtained by heterologous expression of *ant(9)-Ic* in E. coli (Table 1).

### Kinetic characterization of ANT(9)-Ic.

With the resistance activity of ANT(9)-Ic confirmed with a majority of aminoglycosides in both E. coli and its natural host, we next determined the kinetic parameters for ANT(9)-Ic-catalyzed adenylation of spectinomycin. The well characterized ANT(9)-Ib was utilized as a positive control to confirm that our assay allowed detection of adenyltransferase activity. In addition, we also selected streptomycin, tobramycin, gentamicin, and amikacin as a control group. The steady-state kinetic parameters for ANT(9)-Ic and ANT(9)-Ib are summarized in Table 2. Mirroring the resistance phenotypes, ANT(9)-Ic carried out specific adenylation of spectinomycin, while no adenylating activity was detected with streptomycin, tobramycin, gentamicin, or amikacin. As expected, spectinomycin-specific adenylating activity was also detected in the case of ANT(9)-Ib. Furthermore, roughly in proportion to the MICs determined by susceptibility testing (Table 1), the spectinomycin-specific adenylating activity (*k*_cat_/*K_m_*) of ANT(9)-Ib was approximately three to four times greater than that of ANT(9)-Ic, with ANT(9)-Ib displaying higher *k*_cat_ and lower *K_m_* values (Table 2). The reason for this difference may be primarily the different amino acid sequences of the two proteins (34.18% identity), which could influence their basic structure and thus enzyme-substrate binding behaviors during the reaction. Altogether, the data presented above suggest that the ANT(9)-Ic enzyme specifically adenylates spectinomycin and thus consolidates its functional classification as a spectinomycin nucleotidyltransferase.

**TABLE 2:** Kinetic parameters of spectinomycin and other four aminoglycosides for ANT(9)-Ic and ANT(9)-Ib^*a*^

Enzymes^*b*^	Substrates	*K*_cat_ (s^−1^)^*c*^	*K_m_* (μM)^*c*^	*K*_cat_/*K_m_* (M^−1^ s^−1^)
ANT(9)-Ic	SPC	1.2 ± 0.4	44.83 ± 6.2	(2.8 ± 0.6) × 10^4^
	STR	NAAD	NAAD	NAAD
	TOB	NAAD	NAAD	NAAD
	GEN	NAAD	NAAD	NAAD
	AMK	NAAD	NAAD	NAAD
ANT(9)-Ib	SPC	2.6 ± 0.2	33.56 ± 8.1	(8.3 ± 2.1) × 10^4^
	STR	NAAD	NAAD	NAAD
	TOB	NAAD	NAAD	NAAD
	GEN	NAAD	NAAD	NAAD
	AMK	NAAD	NAAD	NAAD

a AMK, amikacin; GEN, gentamicin; NAAD, no adenylating activity detected; SPC, spectinomycin; STR, streptomycin; TOB, tobramycin.

b The proteins were initially modified by a His_6_ tag, which was removed after purification.

c *k*_cat_ and *K_m_* values represent the mean ± standard deviation (SD) of three independent experiments.

### Genetic environment and origin of *ant*(9)-*Ic*.

**FIG 2:**
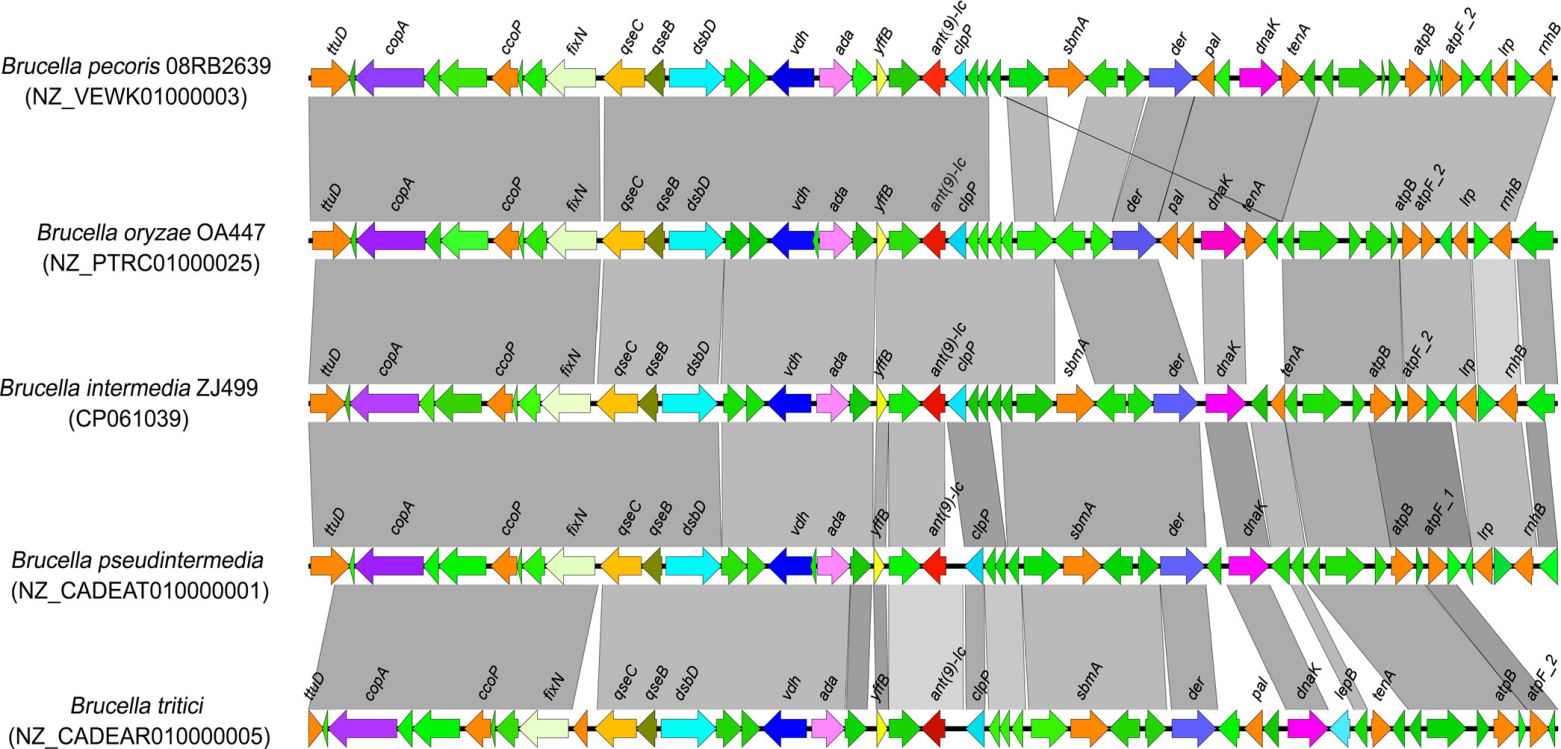
Genetic context of *ant(9)-Ic* in ZJ499 and comparison of the *ant(9)-Ic*-neighboring regions in different Brucella species. Colored arrows indicate open reading frames (ORFs) and the direction of transcription. The *ant(9)-Ic* gene is highlighted in red, and the ORFs encoding hypothetical proteins are colored in green. The shading represent homologous regions between these genetic contexts.

In previous work, we showed that *ant(9)-Ic* was located on the chromosome 1 of B. intermedia ZJ499 (accession no. CP061039). To investigate the genetic context of *ant(9)-Ic*, the structures of the 20-kb flanking region of *ant(9)-Ic* on either side were analyzed and are shown in Fig. 2. The structural gene for *ant(9)-Ic* was preceded by a ATP-dependent Clp protease proteolytic subunit gene, *clpP*. A hypothetical protein coding gene and an ArsC family reductase gene *yffB* were located immediately downstream of *ant(9)-Ic* in the opposite orientation. No evidence of mobile element was found within the 20-kb-long region around *ant(9)-Ic*.

In addition, comparative genomic analysis of the genetic environment of *ant(9)-Ic* in ZJ499 with the *ant(9)-Ic*-bearing fragments in other Brucella species revealed that the region downstream of *ant(9)-Ic* has a highly conserved architecture in both gene content and gene order (Fig. 2). The adjacent region upstream of *ant(9)-Ic* is conserved overall, although some fragment insertion or missing events occurred in certain species (Fig. 2). These observations suggest that *ant(9)-Ic* may be intrinsic in Brucella, which is quite different from other ANT(9)-I coding genes, in which *ant(9)-Ia* was reported to be located on transposon Tn*554* (8, 18), while *ant(9)-Ib* (9), *spw* (11), and *spd* (10) were found on plasmids. Additionally, the finding of *ant(9)-Ic* on chromosome as an intrinsic gene may provide some insight into the natural mechanism of resistance in Brucella, although the primary function of *ant(9)-Ic* in these bacteria remains an open question. It is possible that *ant(9)-Ic* really functions as a spectinomycin resistance gene in nature, which can help the Brucella spp. to ecologically adapt to the spectinomycin-like compounds produced by local soil-dwelling bacteria. Alternatively, the main role of *ant(9)-Ic* is not conferring resistance to antibiotics but being involved in other functions relevant to Brucella physiology and metabolism (19, 20). These issues would require further investigation.

## MATERIALS AND METHODS

### Bacterial strains, plasmids, and culture conditions.

The host strain B. intermedia ZJ499 carrying the novel *ant(9)-Ic* gene was isolated from a patient with non-Hodgkin's lymphoma in 2018, at the Fifth Affiliated Hospital of Wenzhou Medical University in Lishui, Zhejiang, China (16). E. coli DH5α was used as the recipient for transformation in cloning of *ant(9)-I* genes into the pUCP20 vector, and E. coli BL21 was used with the cold shock expression vector pCold I (21) for purification of ANT(9)-I proteins. For screening transformants, ampicillin was used at 100 μg/mL for E. coli, unless noted otherwise. The strains were routinely cultured in LB medium at 37°C, solidified with 1.5% agar when necessary. For pCold I harboring the *lac* operator, IPTG (isopropyl-β-d-thiogalactopyranoside) was added to cultures at an optical density at 600 nm (OD_600_) of 0.6 to 0.8 with a final concentration of 1 mM, and the bacteria were then incubated at 16°C for an additional 16 to 20 h as reported previously (16).

### Antibiotic susceptibility testing.

The MICs of various aminoglycoside antibiotics were determined on Mueller-Hinton plates using the agar dilution method according to the Clinical and Laboratory Standards Institute (CLSI) guidelines. The plates were incubated for 18 to 20 h at 37°C before the results were analyzed. E. coli ATCC 25922 was used as a quality control strain.

### Bioinformatic analyses.

BLAST searches were performed with the nucleotide or protein sequence of ANT(9)-Ic used as a query at the NCBI website (https://blast.ncbi.nlm.nih.gov/Blast.cgi). The molecular weight and pI value of ANT(9)-Ic were predicted using ProtParam (https://web.expasy.org/protparam/). The neighbor-joining phylogenetic tree was generated with MEGA X (22). Analysis of conserved functional domains was performed with InterProScan (https://www.ebi.ac.uk/interpro/). Gene organization diagrams were created with Easyfig (23).

### Gene cloning and construction of *ant(9)-Ic* deletion mutant.

For cloning of *ant(9)-Ic*, the *ant(9)-Ic* ORF and its promoter region were amplified from ZJ499 genomic DNA by PCR and cloned into the pUCP20 vector by restriction enzyme digestion and ligation. The ligation products were introduced into E. coli DH5α by electroporation, and transformants were selected on LB agar containing 100 mg/liter ampicillin. The resulting recombinant plasmids were confirmed by restriction digest analysis and DNA sequencing. For *ant(9)-Ib* cloning, a 1,059-bp DNA fragment containing *ant(9)-Ib* ORF and its promoter region (GenBank accession number M69221) from E. faecalis LDR5537 (9) was custom synthesized by Tsingke Biotechnology (Nanjing, China), and a similar strategy was used for cloning into pUCP20, as described above. All the primers and restriction enzyme sites used for PCR and cloning are listed in Table S1 (available as supplemental material).

To obtain the *ant(9)-Ic* deletion mutant of B. intermedia ZJ499, previously established protocols by allelic exchange using the suicide vector pEX18Gm were followed (16, 24). Briefly, the upstream and downstream regions (~1,000 bp) of *ant(9)-Ic* were amplified and combined by overlap PCR using two sets of primer pairs (Table S1). The resultant fusion PCR product was digested and ligated into pEX18Gm. The plasmid generated was introduced into B. intermedia ZJ499 by biparental mating with E. coli S17-1 λpir for replacement of the wild-type copy of *ant(9)-Ic*, as described previously (16). The resulting deletion mutant missing the 780-nt coding sequence of *ant(9)-Ic* (from bp 1 to 780) was further verified by PCR and sequencing.

### Protein expression and purification.

E. coli BL21 carrying pCold I-*ant(9)-Ic* was used for expression of ANT(9)-Ic as a fusion protein containing an N-terminal His_6_ tag and a thrombin cleavage site as previously described (16). Ni-nitrilotriacetic acid (NTA) affinity chromatography was utilized as an initial purification step, and the His_6_ tag was removed by thrombin cleavage at 25°C for 3 h, followed by a further purification step with an Ni-NTA column to remove the free His_6_ tag. The purified ANT(9)-Ic protein was concentrated with a 10-kDa cutoff ultrafiltration spin column (Sartorius). The size and purity of ANT(9)-Ic were analyzed by SDS-PAGE (12%), and the protein concentration was determined by both spectrophotometric method and the BCA protein assay. ANT(9)-Ib expression and purification were carried out using a similar strategy.

### Enzyme kinetic analysis.

The enzyme activities of ANT(9)-Ic and ANT(9)-Ib were measured using an *in vitro* reaction system as previously described (25). Briefly, adenylation of aminoglycoside antibiotics was detected by a continuous spectrophotometric assay, which couples the production of pyrophosphate (PPi) to the reactions with UDP-glucose pyrophosphorylase, phosphoglucomutase, and glucose-6-phosphate dehydrogenase, and monitoring the formation of NADPH at 340 nm with a UV-visible (UV-VIS) spectrophotometer (U-3900, Hitachi, Japan). The reaction mixture consisted of 50 mM HEPES (pH 7.5), 10 mM MgCl_2_, 0.2 mM UDP-glucose, 0.2 mM 4,4′-dithiodipyridine (DTDP), 2 U/mL UDP-glucose pyrophosphorylase, 20 U/mL phosphoglucomutase, 20 U/mL glucose-6-phosphate dehydrogenase, 50 to 150 μmol purified ANT(9)-Ic or -Ib, 5 mM ATP, and variable concentrations of aminoglycosides. The reactions were initiated by the addition of enzymes and were performed in a final volume of 100 μL in 96-well plates at 37°C. The steady-state kinetic parameters (*k*_cat_ and *K_m_*) were determined by nonlinear regression of the initial reaction rates with the Michaelis-Menten equations using GraphPad Prism 9 (GraphPad Software).

### Data availability.

The complete nucleotide sequence of *ant(9)-Ic* has been deposited in the GenBank database under accession number MZ241296.
