# Supplementary material for: ANT(9)-Ic, a Novel Chromosomally Encoded Aminoglycoside Nucleotidyltransferase from Brucella intermedia
Source: Microbiol Spectr. 2023 Apr 11;11(3):e00620-23. doi: 10.1128/spectrum.00620-23 (PMC10269693; doi:10.1128/spectrum.00620-23)
Supplement: Supplemental file 1 — Table S1. Download spectrum.00620-23-s0001.pdf, PDF file, 0.1 MB [file spectrum.00620-23-s0001.pdf]

Table S1. Primers used in this study

Primers	Sequences (5′ to 3′)	Purpose
Pro-ant(9)-Ic-F	GCTCTAGACAGGCATCGGACATTTTCATCCATG	Cloning of *ant(9)-Ic*
Pro-ant(9)-Ic-R	CGGGATCCGGTTCAGGACACGCTTCTGCAC	
ant(9)-Ic-UP-F	CGGGATCCCGCCATGGTGACGGCGGTGATCTG	Constructing *ant(9)-Ic* deletion plasmid
ant(9)-Ic-UP-R	TGTCGCCAGCAGCGTTGTATATGTTGCTTTTCTGAAAATCTTCAGGCC	
ant(9)-Ic-DN-F	GGCCTGAAGATTTTCAGAAAAGCAACATATACAACGCTGCTGGCGACA	Constructing *ant(9)-Ic* deletion plasmid
ant(9)-Ic-DN-R	CGGAATTCGGATGCCGCGCCGCAGAAAGTCTG	
ant(9)-Ic-DOU-F	TTTACGCATCGGCGCTGCTGTTCGG	Screening *ant(9)-Ic* deletion mutant
ant(9)-Ic-DOU-R	TCAGGGGCAGGCATCGGACATTTTC	
ant(9)-Ic-ORF-thrombin-F	GGAATTCCATATGATGGATAGAAATCACGCAGCAATCC	Cloning of *ant(9)-Ic* into pCold I vector
ant(9)-Ic-ORF-R	CGGGATCCTCAGAAAAGCAGCCGCACCTGATGT	
ant(9)-Ib-ORF-thrombin-F	GGAATTCCATATGGTGAGGAGGATATATTTGAATACATACG	Cloning of *ant(9)-Ib* into pCold I vector
ant(9)-Ib-ORF-R	CGGAATTCTTATAATTTTTTTAATCTGTTATTTAAA	
